# Effect of Metal Content on Ethanol Decomposition over Ni-Co Catalysts Supported on La-Ce Oxides

**DOI:** 10.3390/ma13030759

**Published:** 2020-02-07

**Authors:** Harold R. Vergara, Maria H. Brijaldo, José J. Martinez, Hugo A. Rojas, José Pedraza, Fabio B. Passos, Luiz Pereira da Costa, Daniela Gonzalez-Vera, Paula Osorio-Vargas

**Affiliations:** 1 Grupo de Catálisis (GC-UPTC), Universidad Pedagógica y Tecnológica de Colombia UPTC, Avenida Central del Norte, Vía Paipa, Tunja 150001, Colombia; 2 Grupo de Investigación en Gestión Administrativa y Empresarial Sostenible (GIGAS), Universidad Pedagógica y Tecnológica de Colombia UPTC, Avenida Central del Norte, Vía Paipa, Tunja 150001, Colombia; 3 Laboratório de Reatores, Cinética e Catalise (RECAT), Departamento de Engenharia Química e de Petróleo, Universidade Federal Fluminense, Niterói 24210-240, Brazil; 4 Graduate Program in Science and Technology for Amazon Resources (PPGCTRA)-Institute of Exact Sciences and Technology (ICET/UFAM), ItcRua: Nossa Senhora do Rosario, 3863, Tiradentes, Itacoatiara-Amazonas 69103-208, Brazil; 5 Grupo de Investigación en Fotocatálisis y Estado Sólido (GIFES), Escuela de Química, Universidad Tecnológica de Pereira, 660003 Pereira, Colombia; 6 Laboratory of Thermal and Catalytic Processes (LPTC), Department of Chemical Engineering, University of Bío-Bío, Concepción 4081112, Chile

**Keywords:** bimetallic catalysts, Ni-Co, ethanol decomposition, perovskite precursor, supports of La-Ce oxides

## Abstract

The search for catalysts with features that can improve coke resistance and decrease byproduct formation is a current goal in H_2_ production from renewable sources. In this work, the effect of the presence of Ni nanoparticles over Co/La-Ce oxides on the ethanol decomposition reaction was studied. Catalysts were synthetized using as precursor a La_0.8_Ce_0.2_Ni_x_Co_1-x_O_3_ perovskite-type material to ensure a low segregation of phases and a high dispersion of metals. After reduction at 873 K, the perovskite structure was destroyed, and metal Co-Ni particles were supported over a lanthanum-cerium oxide. The materials were characterized by different techniques before and after reaction. Solids exhibited metal particle sizes between 5 and 15 nm demonstrating the advantages of the preparation method to obtain Ni-Co alloys. Although the results of adsorption of ethanol followed by diffuse reflectance infrared fourier transformed spectroscopy (DRIFTS) showed acetate species strongly adsorbed on the catalyst’s surface, the material (Ni_0.7_Co_0.3_/La_0.8_Ce_0.2_) with the lowest particle size was the most stable system leading to the lowest amount of carbon deposits during ethanol decomposition. This catalyst showed the better performance, with a higher ethanol conversion (98.4%) and hydrogen selectivity (75%). All catalysts exhibited carbonaceous deposits, which were an ordered and disordered carbon phase mixture.

## 1. Introduction

Currently, fossil fuels such as coal, oil, and natural gas are the conventional energy sources. Their continuous use can negatively affect the environment. For this reason, sustainable and renewable energy sources are necessary in order to reduce pollutant emissions and preserve the environment. Within these renewable energies, H_2_ seems to be a promising fuel which can be obtained from several byproducts originated from biomass conversion. For instance, ethanol, which can be produced by simultaneous saccharification and fermentation (SSF) [1], exhibits interesting features as a H_2_ source. Several chemical processes such as ethanol steam reforming (ESR), partial oxidation (POE), oxidative steam reforming (OSPE), and catalytic ethanol decomposition (ED) have been evaluated for H_2_ production. In particular, ED (Equation (1)) shows interesting characteristics since it is an endothermic reaction (∆H° = 49.6 kJ mol^−1^) requiring less energy than the more widely studied ESR process (∆H° = 173.5 kJ mol^−1^). However, as is the case with ESR, ED exhibits some drawbacks related to the existence of simultaneous reactions such as the Boudouard reaction (Equation (2)) and methane decomposition (Equation (3)), which could lead to catalyst deactivation by deposition of carbonaceous species [2].

(1) $$CH_{3}CH_{2}OH\;\to \;CH_{4}+CO+H_{2}\;\;\Delta H{}^{\circ}=\mathrm{kJ}\;{\mathrm{mol}}^{-1}$$

(2) $$2CO\;\to \;CO_{2}+C\;\;\Delta H{}^{\circ}=-172.6\;\mathrm{kJ}\;{\mathrm{mol}}^{-1}$$

(3) $$CH_{4}\;\to \;C+2H_{2}\;\;\Delta H{}^{\circ}=74.9\;\mathrm{kJ}\;{\mathrm{mol}}^{-1}$$

At low temperatures, where reaction (2) is more important, the catalyst can be deactivated through the formation of amorphous carbon deposits covering the metal particles. When the reaction is carried out at high temperatures (>600 °C), reaction (3) becomes important, participating as well in the formation of carbonaceous species. However, on Ni and Co particles, this deposited carbon nucleates the growth of carbon filaments do not lead to total catalyst deactivation, since these filaments can push off the metal particles from the support leaving the metal accessible to reactants and exhibiting a minor deactivating effect [3].

Therefore, one of the main drawbacks to overcome so that this kind of process can be considered a viable route for the clean production of H_2_ is related to the stability and resistance of the catalysts toward coke formation. This can be achieved by a catalyst tailored to reduce coke formation or favor its rapid conversion into gaseous byproducts. In the literature, several approaches have been proposed to achieve this goal: (i) use of a support which can help carbon removal, (ii) particle ensemble size control, and (iii) addition of a second metal in order to improve modifications in catalytic performance. Regarding this last approach, a recent study highlighted a marked improvement in catalyst stability when 1%Rh was added to 10%Ni/Al_2_O_3_ promoted with La_2_O_3_ and CeO_2_ [4]. In that case, deposition of carbon decreased by 560 times compared with the Ni catalyst. In contrast, controlling the ensemble size seeks to inhibit carbon formation since a critical ensemble size is necessary for its formation. ESR measurements performed by Rostrup-Nielsen [5] showed that this reaction needs ensembles of 3 or 4 atoms, whereas carbon formation requires 6 or 7 atoms. Hence, particle size can influence carbon nucleation. In the search to control the particle ensemble size, catalysts using layered double hydroxide (LDH) and perovskite-type structures (ABO_3_) as precursors have been investigated [6,7,8,9,10]. Reduction of LDH or perovskite-type structures leads to high dispersion on corresponding oxides. Manukyan et al. [7] obtained a Ni/Al_2_O_3_ system from the reduction of a Ni-Al hydrotalcite-type material. Those authors evaluated the deactivation of Ni-Al_2_O_3_-LDH and Ni/Al_2_O_3_ catalysts in the ethanol decomposition reaction. The XPS results showed that the amount of carbon increased up to 48% after 100 h time on stream (TOS) and 2 h TOS for Ni-Al_2_O_3_-LDH and Ni/Al_2_O_3_, respectively, which was related to the load and size of the metal particles. The 80%Ni-Al_2_O_3_-LDH catalyst exhibited nickel with small particle sizes having diameters around 3–5 nm, whereas Ni particles on the 10%Ni/Al_2_O_3_ catalyst were around 7–15 nm. Gallego et al. [9] also obtained Ni nanoparticles (15 nm, mean diameter) highly dispersed on La_2_O_3_ when prepared by reduction of LaNiO_3_ perovskite. This catalyst was very active for the ethanol decomposition reaction to H_2_ production and carbon nanotubes. In general, an important number of studies based on supported metal catalysts (Ni, Co, and Fe) on different supports such as La_2_O_3_, CeZrO_2_, MgO, Al_2_O_3_, CeO_2_, and graphite for H_2_ production by ethanol decomposition have been published [7,8,9,11,12,13,14,15,16,17,18,19,20]. In fact, some studies have been focusing on both H_2_ production and carbon nanotube (CNT) production instead of the removal of carbonaceous deposits on metallic particles [9,15,16,17,18,19].

Taking into account the above-mentioned strategies to improve catalyst activity and stability by decreasing carbon deposition, the goal of this research was to study the effect of a second metal presence (Ni) in Co/La-Ce oxide catalysts prepared from a perovskite-type La_0.8_Ce_0.2_Ni_x_Co_1-x_O_3_ precursor for the ethanol decomposition reaction. This preparation method may favor the formation of small metal particles. The active phase was modified with different Ni and Co amounts to study a possible synergistic effect between both metals, since each one has already exhibited a good performance individually on catalytic ethanol decomposition. Their oxophilic nature favors O-H bond activation of ethanol [21]. Mixed La-Ce oxides were selected in the search for supports that increase catalyst stability, as both oxides have been used with excellent results in this type of reaction [9,20,22]. The lattice oxygen of CeO_2_ can react with adsorbed hydrocarbons on surface, producing synthesis gas and thus preventing the formation of carbon species from hydrocarbon decomposition reactions (C_m_H_m_ → nC + m/2H_2_) [20]. The effect of Co and Ni metal amounts on the physico-chemical catalyst properties, H_2_ production, and carbon depositions in the reaction of ethanol decomposition at a temperature range from 303 K to 973 K was evaluated.

## 2. Materials and Methods

### 2.1. Catalyst Synthesis and Characterization

Perovskites were prepared by the polymeric precursor method or Pechini method [23] that allows, in general, to obtain a homogeneous organometallic polymer where metal is located in the polymer’s main chain. The cation salts (La(NO_3_)_3_·6H_2_O, Ce(NO_3_)_3_·6H_2_O, NiCl_2_·6H_2_O, and CoCl_2_·6H_2_O) were mixed with the suitable amounts to obtain the La_0.8_Ce_0.2_Ni_x_Co_1-x_O_3_ (x = 0, 0.1, 0.4, and 0.7) perovskites and then were added to a solution of citric acid (CA) and ethylene glycol (EG). The ratio molar of CA:EG:metal ions was of 6:3:1. The prepared solution was slowly heated to 353 K under constant stirring until gel formation. The resulting resin containing cations was further heated to 623 K for 3 h yielding a fine powder. This material was calcined at 773 K for 1 h and then at 973 K for 6 h (5 K min^−1^). Perovskite precursors were subsequently transformed to metal catalysts supported on oxides of Ce and La, by reduction under H_2_ flow (30 mL min^−1^) at 873 K for 2 h. After this, the samples were passivated, using an O_2_(1%)/He mixture for 0.5 h at N_2_ liquid temperature to carry out some characterization techniques.

The textural properties were obtained from N_2_ physisorption analysis at 77 K in an ASAP 2020 Micromeritics instrument (Micromeritics Instrument Corp., Norcross, GA, USA). The passivated catalysts were treated at 593 K for 12 h and outgassed at 393 K for 2 h under N_2_ flow. The surface area values were determined from the Brunauer-Emmett-Teller model (BET model) and the pore-size distributions were calculated from the N_2_ adsorption branch using the Barrett, Joyner, and Halenda method (BJH method). X-ray diffraction (XRD) analyses were performed on a Rigaku Miniflex II instrument (Rigaku Corporation, Tokyo, Japan) with Cu K radiation (kα = 0.154 nm) using a 0.05° step size. H_2_ chemisorption was studied to determine metallic dispersion assuming an adsorption stoichiometry of H/M = 1. A typical protocol was followed, the solids dried at 473 K for 1 h (30 mL min^−1^) were posteriorly reduced under in situ H_2_ (30 mL min^−1^) for 2 h at 873 K in a Micromeritics ASAP 2020 instrument (Micromeritics Instrument Corp.). Afterward, the surface was cleaned under He flow at the same temperature for 1 h at 873 K. Then, the systems were brought to room temperature and total hydrogen isotherms were acquired. To determine the total acidity, the solids previously purged were submitted to adsorption of ammonia saturated at 383 K in 10%NH_3_/He flow (30 mL min^−1^). Temperature-programmed desorption was performed using a heating rate of 10 K min^−1^, from room temperature until reaching 1173 K.

Temperature-programmed reduction (TPR) analysis was performed in a Micromeritics Autochem 2920 analyzer (Micromeritics Instrument Corp.). The catalysts (100 mg) were dried under He flow at 573 K for 0.5 h and the TPR profiles were obtained by heating the samples under a 10% H_2_/Ar flow (30 mL min^−1^) from 298 to 1173 K (10 K min^−1^). Transmission electron microscopy (TEM) micrographs were obtained on a Jeol JEM 2100 HTP apparatus (Jeol Ltd., Tokyo, Japan) operating at 200 kV. The samples were suspended in isopropanol and ultrasonicated for 0.5 h. A drop of the suspension was dropped onto carbon-coated copper grids, allowing the solvent to evaporate at 353 K in vacuum. X-ray photoelectron spectroscopy (XPS) analysis was carried out in a Thermo Scientific Escalab 250 XI model (Thermo Fisher Scientific, Waltham, MA, USA) with monochromatic Al Kα radiation. The measurements were performed at room temperature and a pressure of 7.8 × 10^−8^ mbar. The scans were measured at a pass energy of 25 eV and a step size of 0.05 eV. In all experiments, Ar^+^ ion etching was used and the conditions were as follows: ion energy, 3000 eV; etch time, 60 seconds; and current, medium. Binding energies (BE) were calibrated to the C1s peak (284.6 eV) and the spectra were examined with CasaXPS software (version 2.3.16 PR 1.6).

### 2.2. Catalytic Ethanol Decomposition Experiments

Ethanol decomposition was performed in gas phase in a continuous-flow, atmospheric- pressure reactor. Prior to reaction, the catalyst samples (100 mg) were dried at 473 K under He and reduced with H_2_ at 873 K for 2 h, followed by heating until reaching 973 K under He flow. The reactant mixture (5% ethanol and 95% He, 100 mL min^−1^) was acquired by bubbling He through a saturator containing ethanol at 293 K. The reactions were studied under isothermal conditions at 873 K for 13 h. The ethanol and decomposition products were followed by gas chromatography (Varian 3400, Palo Alto, CA, USA) using Carboxen 1010 plot and Porabond Q capillary columns. Ethanol conversion (X) and hydrogen selectivity (S) were determined employing the following equations, where *y_j_* is the molar fraction of compound *j* in the exit stream:

$$\begin{array}{c} X_{C_{2}H_{5}OH}=\frac{y_{CO}+y_{CH_{4}}+y_{CO_{2}}+2y_{C_{2}H_{4}O_{}}}{2y_{C_{2}H_{5}OH}+y_{CO}+y_{CH_{4}}+y_{CO_{2}}+2y_{C_{2}H_{4}O_{}}}\times 100\\ S_{H_{2}}=\frac{y_{H_{2}}}{y_{H_{2}}+2y_{CH_{4}}+y_{H_{2}O}+2y_{C_{2}H_{4}O_{}}}\times 100 \end{array}$$

### 2.3. In Situ Diffuse Reflectance Infrared Spectroscopy (DRIFTS) Measurements

Diffuse reflectance infrared spectroscopy (DRIFTS) analysis was carried out in an FTIR spectrometer (VERTEX 70–Bruker, Billerica, MA, USA) with a DRIFTS cell (Harrick Scientific, HVC-DRP-4, New York, NY, USA) and a praying mantis mirror assembly (Harrick Scientific). Prior to C_2_H_5_OH-DRIFTS analysis, the passivated catalysts were dried at 473 K under He flow for 30 min, reduced at 673 K under H_2_ flow (30 mL min^−1^), and cooled under He flow (30 mL min^−1^) until reaching 303 K. At this temperature, a background interferogram was collected, as well as at 573, 473, 373, and 303 K. The adsorption of ethanol was conducted at 293 K using an ethanol/He mixture, which was obtained by flowing He through a saturator ethanol at 293 K. After adsorption, the samples were purged under He flow and a new interferogram was taken, which related to the background reference to acquire the spectrum of adsorbed ethanol.

### 2.4. Spent Catalyst Characterization

The amount of the coke deposited on the spent catalysts was quantified by thermogravimetry (TGA/DTA, Shimadzu-60H, Kyoto, Japan) through the mass loss during the analysis. The samples were dried up to 423 K for 1 h in He flow (30 mL min^−1^) to remove water and physisorbed species on the catalyst surface and cooled under He flow until reaching 303 K. At this temperature, TGA was carried out under airflow until reaching 1200 K (10 K min^−1^). Quantification of carbon was calculated according to the following equation:

C = m_coke_/m_spent catalyst_

The spent catalysts were studied by Raman spectroscopy using a Confocal Raman Microscope alpha300 (Witec, Ratingen, Germany) with a 50× objective lens, a laser with 532 nm wavelength, and 450 scans.

## 3. Results

### 3.1. Catalyst Characterization 

**Figure 1:**
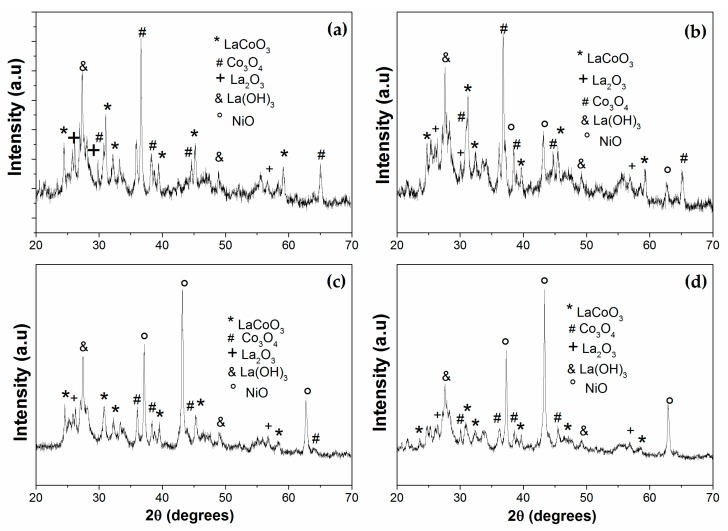
X-ray diffraction patterns of calcined catalysts: (**a**) Co/La_0.8_Ce_0.2_; (**b**) Ni_0.1_Co_0.9_/La_0.8_Ce_0.2_; (**c**) Ni_0.4_Co_0.6_/La_0.8_Ce_0.2_; and (**d**) Ni_0.7_Co_0.3_/La_0.8_Ce_0.2_.

**Figure 2:**
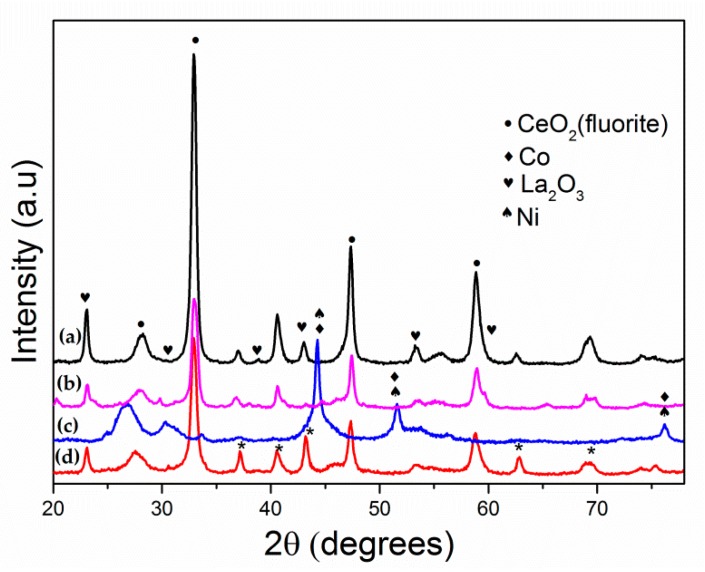
X-ray diffraction patterns of passivated catalysts: (a) Ni_0.7_Co_0.3_/La_0.8_Ce_0.2_, (b) Ni_0.4_Co_0.6_/La_0.8_Ce_0.2_, (c) Ni_0.1_Co_0.9_/La_0.8_Ce_0.2_, and (d) Co/La_0.8_Ce_0.2_.

Diffractograms of passivated catalysts synthesized by the Pechini method are shown in Figure 1. The fresh catalyst with x = 0 showed six diffraction angles (2θ) at 23.2, 32.9, 33.3, 40.7, 47.5, and 59.0° related to the (012), (110), (104), (202), (024), and (214) respective crystal planes of rhombohedral LaCoO_3_ (JCPDS 48-0123). For the Ni-containing catalyst, these peaks are slightly displaced, which could be a consequence of the possible insertion of Ni into the structure. In addition, a diffraction line at 2θ ~ 32.7° attributed to the LaNiO_3_ structure [24] was mainly detected for the La_0.8_Ce_0.2_Ni_0.7_Co_0.3_ precursor. This result is not surprising given the greater amount of nickel in this precursor. Strong peaks corresponding to Co_2_O_3_, La(OH)_3_, and NiO were also observed. Peaks detected at 31.8, 36.2, 44.9, 59.5, and 65.5° are due to planes (2 2 0), (3 1 1), (4 0 0), (5 1 1), and (4 4 0), respectively, (JCPDS 65-3103) of the Co_2_O_3_ cubic phase, while those observed at 2θ of 15.8, 28.1, and 49.1° (JCPDS 00-036-1841) are characteristic of La(OH)_3_. The diffraction lines corresponding to NiO (2θ = 37.2, 43.3, and 62.9° JCPDS 01-073-1519) appeared mainly in the precursor with higher nickel content (La_0.8_Ce_0.2_Ni_0.7_Co_0.3_) although slightly shifted. The presence of these latter phases is an indication that there was possibly an excessive amount of cations that led to the segregation of these species, as has been reported by other authors [25,26,27]. The passivated Ni_0.7_Co_0.3_/La_0.8_Ce_0.2_, Ni_0.4_Co_0.6_/La_0.8_Ce_0.2_, and Co/La_0.8_Ce_0.2_ catalysts (Figure 2) exhibited clear signals indexed at 2θ = 28.6° (1 1 1), 33.1° (2 0 0), 47.6° (2 2 0), and 56.5° (3 1 1) (JCPDS 081-0792) attributed to a fluorite-type structure of CeO_2_. Similarly, these diffractograms revealed the presence of peaks corresponding to La_2_O_3_ at 26.1, 29.1, 29.9, 39.64, 46, and 52° (JCPDS 01-074-2430). The La(OH)_3_ phase was also detected after the reduction stage. Diffraction lines attributable to Co_2_O_3_ were still observed in the Co/La_0.8_Ce_0.2_ catalyst after reduction, showing that this species was hardly reducible and probably the presence of Ni (Ni_0.7_Co_0.3_/La_0.8_Ce_0.2_, Ni_0.4_Co_0.6_/La_0.8_Ce_0.2_, and Ni_0.1_Co_0.9_/La_0.8_Ce_0.2_) favored its reduction. However, XRD signals were only observed for Ni_0.1_Co_0.9_/La_0.8_Ce_0.2_ systems due to metallic Co (2θ = 44.3, 51.8, and 76.3°) and Ni (2θ = 44.5, 51.8, and 76.5°). It is possible that these peaks were not detectable for the other catalysts due to a high dispersion of these species, as confirmed by H_2_ chemisorption and TEM (Table 1).

**Figure 3:**
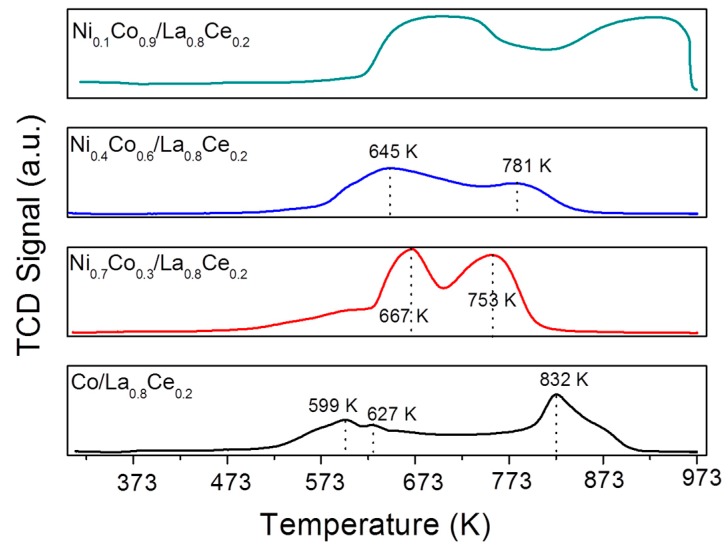
Temperature-programmed reduction (TPR) profiles of the prepared catalysts.

Figure 3 shows the H_2_-TPR profiles of the supported Ni-Co catalysts. The reduction profile of all oxidized catalysts exhibited two peaks in the range between 470–700 K and 700–923 K with different maximum temperatures between them.

**Figure 4:**
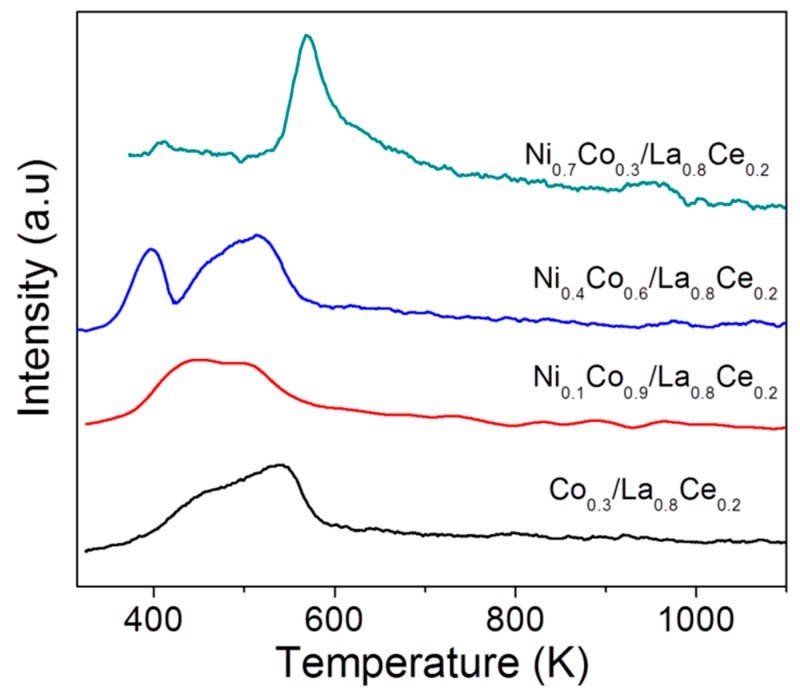
NH_3_-TPD profiles of the prepared catalysts Co_x_Ni_1-x_/La_0.8_Ce_0.2_ for acidity determination.

The first peak can be related to the reduction of Co^3+^ to Co^2+^, at the same time as the Ni^3+^ to Ni^2+^, coming from the perovskite structure, as has already been reported [26,27,28]. Subsequently, the second peak corresponds to the reduction of Co^2+^ to Co^0^ and/or Ni^2+^ to Ni^0^ of the same structure. When the amount of Ni increases, the reduction temperature is shifted to lower temperatures (Ni_0.7_Co_0.3_/La_0.8_Ce_0.2_ and Ni_0.4_Co_0.6_/La_0.8_Ce_0.2_), which can be ascribed to the reduction of Ni^2+^ to Ni^0^ into the nickel oxide phase [26,27,29] found by XRD. In the Co/La_0.8_Ce_0.2_ catalyst, the lowest observed reduction temperature (~573 K) can also be associated with the reduction of Co_3_O_4_→CoO→Co [29]. Based on XRD and TPR results, after reduction at 873 K, the perovskite structure was destroyed, and metal Co-Ni particles were supported over lanthanum-cerium oxides. Total acidity of the samples was determined by NH_3_-TPD (Figure 4), and the results are shown in Table 1.

Generally, the strength of acid sites is classified depending on the temperature in which the desorption peaks appear as follows: weak (<473 K), medium (between 473 and 673 K) and strong (>673 K). The Co/La_0.8_Ce_0.2_, Ni_0.1_Co_0.9_/La_0.8_Ce_0.2_, and Ni_0.4_Co_0.6_/La_0.8_Ce_0.2_ catalysts exhibited some signals corresponding to weak and intermediate acid sites, whereas the Ni_0.7_Co_0.3_/La_0.8_Ce_0.2_ system showed only intermediate acid sites.

**Figure 5:**
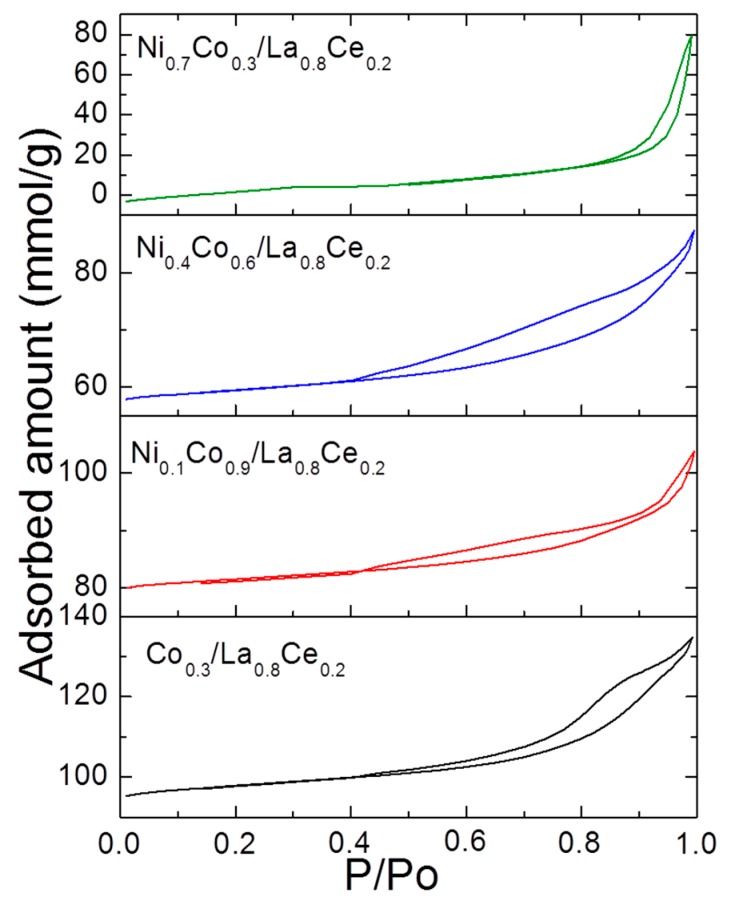
N_2_ adsorption–desorption isotherms for Ni_1-x_Co_x_/La_0.8_Ce_0.2_ catalysts.

Table 1 also shows the textural properties of catalysts studied. An increase in the cobalt loading in the bimetallic catalysts seems to decrease the surface area, thus Ni_0.7_Co_0.3_/La_0.8_Ce_0.2_ exhibited the highest surface (33 m^2^ g^−1^), whereas Ni_0.1_Co_0.9_/La_0.8_Ce_0.2_ had the lowest surface area (14 m^2^ g^−1^). The low surface area of the latter was probably due to the blocking of the support pores by cobalt particles. Figure 5 shows the N_2_ adsorption–desorption isotherms for all catalysts. The isotherms for the Ni-Co catalysts were type IV with H3 hysteresis, suggesting slit-shaped pores [30].

**Figure 6:**
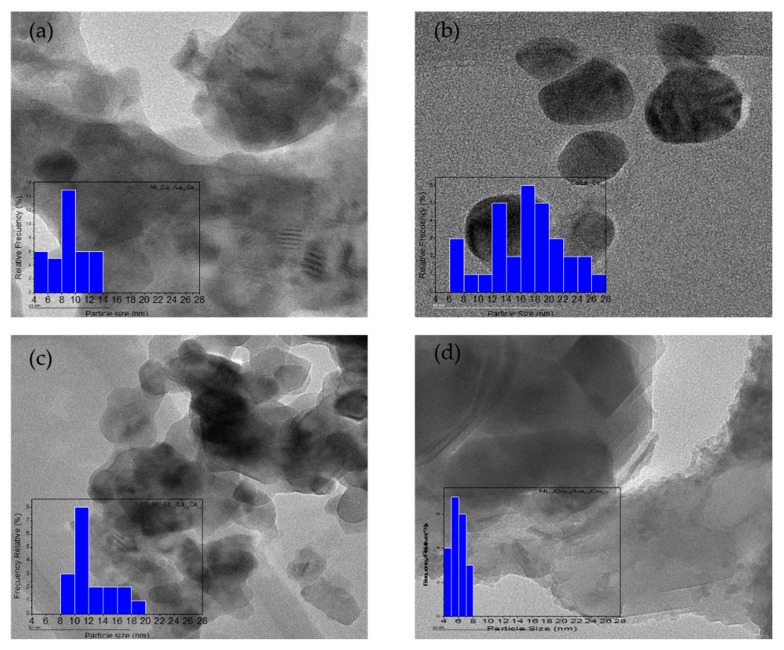
TEM micrographs of Ni_1-x_Co_x_/La_0.8_Ce_0.2_ catalysts: (**a**) Ni_0.7_Co_0.3_/La_0.8_Ce_0.2_; (**b**) Co/La_0.8_Ce_0.2_; (**c**) Ni_0.1_Co_0.9_/La_0.8_Ce_0.2_; and (**d**) Ni_0.4_Co_0.6_/La_0.8_Ce_0.2_.

TEM micrographs with their corresponding histograms are presented in Figure 6. Particle sizes as determined by TEM are shown in Table 1. The presence of both metals had an effect on particle size—as the nickel content increased, the particle size decreased—as has been reported previously [31]. This could be an indication of interaction between metals. As the histograms show, for catalysts with low or no Ni content, a wide size distribution was observed, contrary to that observed for Ni > 0.1. The presence of both metals resulted in small particle sizes in a narrow distribution. These results were confirmed by H_2_ chemisorption measurements.

**Figure 7:**
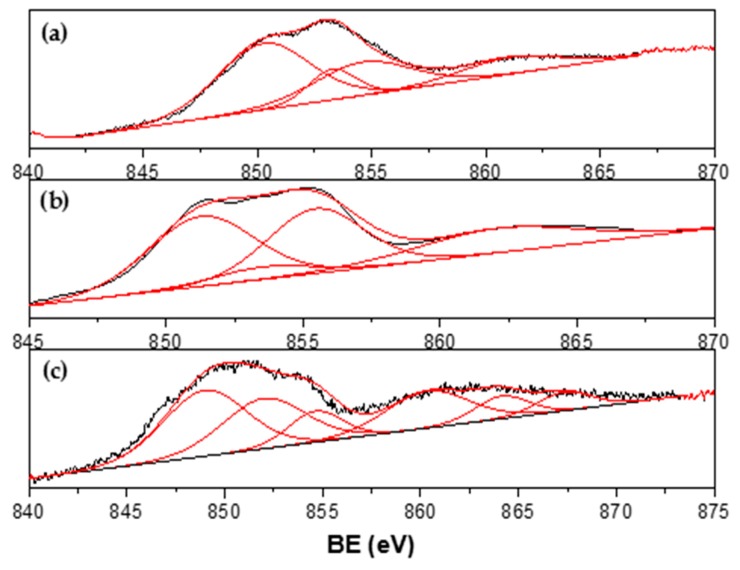
Ni 2p XPS spectra of (**a**) Ni_0.1_Co_0.9_/La_0.8_Ce_0.2_; (**b**) Ni_0.4_Co_0.6_/La_0.8_Ce_0.2_; and (**c**) Ni_0.7_Co_0.3_/La_0.8_Ce_0.2_.

**Figure 8:**
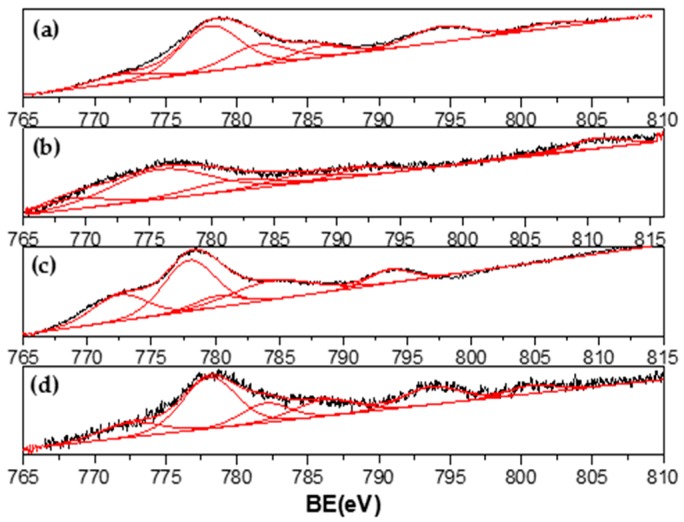
Co 2p XPS spectra of (**a**) Co/La_0.8_Ce_0.2_; (**b**) Ni_0.1_Co_0.9_/La_0.8_Ce_0.2_; (**c**) Ni_0.4_Co_0.6_/La_0.8_Ce_0.2_; and (**d**) Ni_0.7_Co_0.3_/La_0.8_Ce_0.2_.

**Figure 9:**
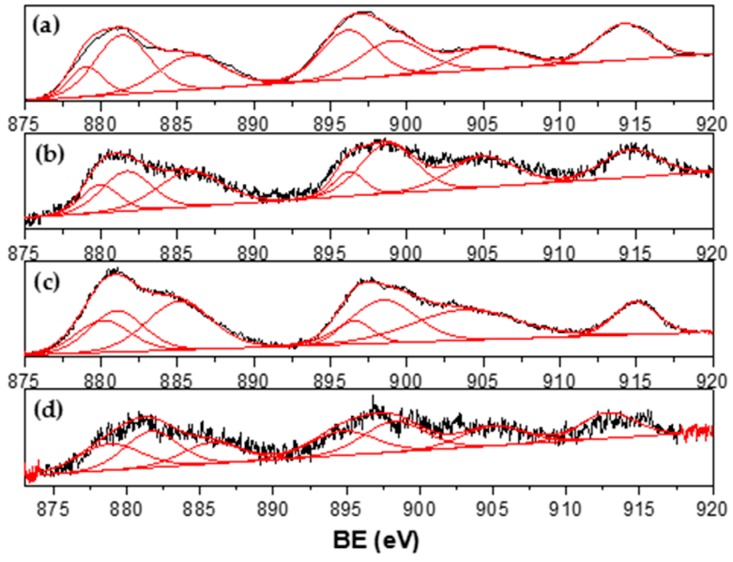
Ce 3d_3/2,5/2_ XPS spectra of supported Ni_1-x_Co_x_/La_0.8_Ce_0.2_ catalysts. (**a**) Co; (**b**) Ni_0.1_Co_0.9_; (**c**) Ni_0.4_Co_0.6_; and (**d**) Ni_0.7_Co_0.3_.

**Figure 10:**
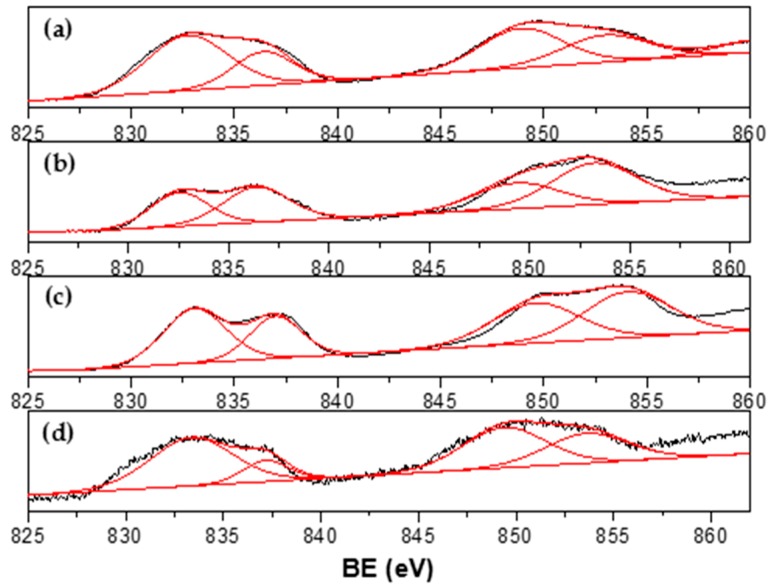
La 3d spectra of Ni_1-x_Co_x_/La_0.8_Ce_0.2_ catalysts. (**a**) Co; (**b**) Ni_0.1_Co_0.9_; (**c**) Ni_0.4_Co_0.6_; and (**d**) Ni_0.7_Co_0.3_.

The XPS spectra for Ni_1-x_-Co_x_ catalysts are shown in Figure 7, Figure 8, Figure 9 and Figure 10. Signal deconvolution of the Ni 2p_3/2_ core-level region (Figure 7) led to four main peaks, centered at 850.8, 853.1, 855.1, and 861.4 eV. The peaks at ~853.1 eV (main peak) and 861.4 eV (satellite peak) correspond to NiO, while the peak at 855.1 was assigned to Ni(OH)_2_ [32,33]. According to the literature, the Ni metal 2p_3/2_ peak position is found at around 852.3 ± 0.4 eV [31,34]; however, in this study, this peak underwent a shift around 0.8–1.8 eV. This result could be related to a possible Ni–Co interaction. For Co, deconvolution curves of Co2p binding energy range from 770 eV to 810 eV and are shown in Figure 8.

Signals obtained are consistent with Co metallic and Co in Co^2+^ and Co^3+^ oxidation states, as has been reported [34,35,36,37]. A peak detected around 777.6 eV could be assigned to metallic Co but shifted approximately 0.6 eV from the reported value [34,35,36,37], which confirms the results found for Ni XPS. Co^2*^ and/or Co^3+^ species were identified by the presence of peaks around 780 eV and the characteristic strong satellite peak at around 786 eV ascribed to CoO. XPS results confirmed the presence of metallic particles of Ni and Co and may suggest an interaction between them.

Ce 3d_3/2,5/2_ spectra (Figure 9) are composed of multiplets corresponding to the spin-orbit split 3d_5/2_ and 3d_3/2_ core holes for Ce^4+^ ions. The binding energy located around 917 and 898.3 eV were attributed to a Ce 3d^9^4f^0^ O 2p^6^ final state, being the first peak attributed to Ce^4+^ ions. The other peaks found at 901.3, 882.7, 907.3, and 888.5 eV are due to Ce 3d^9^4f^2^ O 2p^4^ and 3d^9^4f^1^ final states [38,39]. These results indicate that the surface of the catalysts are composed of CeO_2_ oxides; however, a broadening or shoulder observed in spectra at about 885.3 and 903.8 eV reveals that Ce^3+^ ions were also present on the support surface [40]. These peaks are the result of a Ce 3d^9^4f^1^ O 2p^6^ final state [39]. The La 3d spectra for Ni_1-x_Co_x_/La_0.8_Ce_0.2_ catalysts (Figure 10) consist of two doublets corresponding to La 3d_5/2_ and 3d_3/2_ core levels observed at 833.1 and 849.5 eV, respectively, and the satellite split to 4.2 eV reported for La_2_O_3_ [41,42]. XPS results confirmed that the support surface is composed of La_2_O_3_ and CeO_2_ oxides.

### 3.2. Evaluation of Adsorbed Species on the Catalyst Surface During Ethanol Decomposition by DRIFT

**Figure 11:**
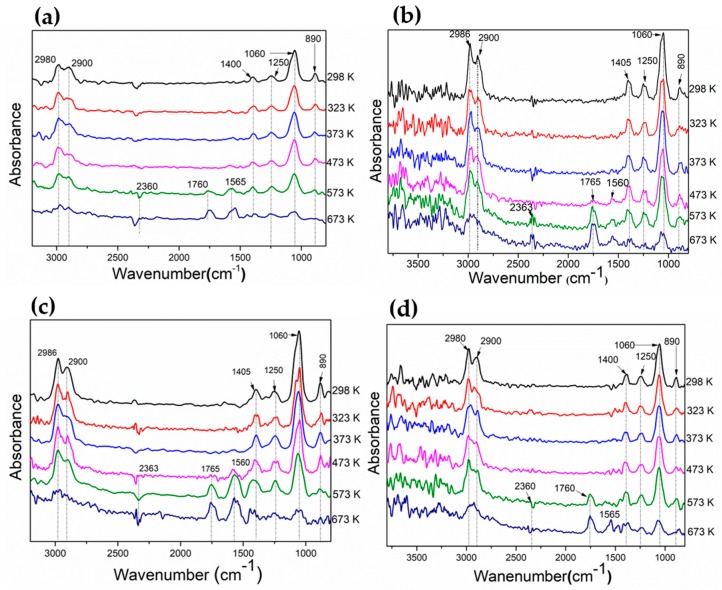
Diffuse reflectance infrared spectroscopy (DRIFT) spectra of ethanol decomposition collected for supported Ni_1-x_Co_x_/La_0.8_Ce_0.2_ catalysts. (**a**) Co; (**b**) Ni_0.1_Co_0.9_; (**c**) Ni_0.4_Co_0.6_; and (**d**) Ni_0.7_Co_0.3_.

Adsorbed species on the catalyst surface during ethanol decomposition from 303 to 673 K were evaluated by DRIFT spectroscopy (Figure 11). Ethanol adsorbed through ethoxy species on the surface catalysts. The ethoxy species is represented by bands at 2980 cm^−1^ (anti-symmetric ν_as_ CH_3_), 2900 cm^−1^ (symmetric ν_s_ CH_3_), 1060 cm^−1^ (symmetric ν_s_ C–O), and 1400 cm^−1^ (δ_s_CH_3_), and its formation on the catalyst surface is due to the scission of the O-H bond. When the temperature rose, band intensities were reduced probably due to the transformation of ethoxy species. At 473 K, bands at 1765 cm^−1^ and 1560 cm^−1^ corresponding to ν_s_(CO) and ν_as_(COO) appeared, which can be due to dehydrogenation from ethoxy species leading to adsorbed acetaldehyde and/or acetate. Acetaldehyde may be dehydrogenated to acetyl species, and this intermediate may undergo support-induced oxidation to acetate species by both O_support_ or OH_support_, as has been reported [43,44,45]. This can be favored on supports of redox oxides as CeO_2_.

(4) $$CH_{3}CH_{2}O_{ads}\;\to \;CH_{3}CHO_{ads}+H_{2}$$

(5) $$CH_{3}CH_{2}O_{ads}\;\to \;CH_{3}CO_{ads}\;\to \;CH_{3}COO_{ads}$$

As temperature rises, band intensities associated with ethoxy species drop, suggesting their conversion with the concomitant rising of bands associated with acetaldehyde and/or acetates. As can be observed for Ni_0.1_Co_0.9_ and Ni_0.4_Co_0.6_, these species rose at lower temperatures. On the other hand, acetate formation was more favored on Co and Ni_0.4_Co_0.6_, which was confirmed by the low intensities of signals for C–H stretching (2800–3050 cm^−1^). Conversely, the band attributed to acetaldehyde was more pronounced for Ni_0.1_Co_0.9_ and Ni_0.7_Co_0.3_. Both acetaldehyde and acetates can be decomposed leading to the formation of CO_2_/CO and CH_4_ (Equations (6) and (7)), and the bands at 2363 cm^−1^ and 3017 cm^−1^, respectively, confirm their presence. Since the goal is that ethanol decomposition should address the selective generation of H_2_, the formation and decomposition of acetaldehyde and acetates are undesirable reactions. In addition, acetates can remain adsorbed on catalyst-blocking catalytic sites [22]. All catalysts showed signals attributable to these species even at high temperature, although the acetate signal on Ni_0.7_Co_0.3_ was less significant, which could point at a higher stability of this catalyst.

(6) $$CH_{3}CHO\;\to \;CH_{4}+CO_{ads}$$

(7) $$CH_{3}COO_{ads}\;\to \;CH_{3\left(ads\right)}+CO_{2}{}_{ads}$$

### 3.3. Ethanol Decomposition Catalytic Tests

The initial rate of reaction (−*r_a_*)_0_ for the ethanol decomposition was determined considering an irreversible, pseudo-first-order chemical reaction under isothermal conditions (973 K). The results are shown in Table 2. These rate values decreased in the following sequence: Ni_0.7_Co_0.3_/La_0.8_Ce_0.2_ > Ni_0.4_Co_0.6_/La_0.8_Ce_0.2_ > Ni_0.1_Co_0.9_/La_0.8_Ce_0.2_ > Co/La_0.8_Ce_0.2._ This behavior indicated that catalysts with high Ni content had a greater capacity to decompose ethanol than the systems having a low Ni content or no Ni. A different behavior was observed when the turnover frequency (TOF) was calculated; Co/La_0.8_Ce_0.2_ and Ni_0.1_Co_0.9_/La_0.8_Ce_0.2_ catalysts were the most active systems for ethanol decomposition at initial or clean conditions. The Weisz–Prater criterion (C_WP_), ethanol conversion, and hydrogen selectivity for the Ni_1-x_Co_x_/La_0.8_Ce_0.2_ catalysts were calculated, and the results are shown in Table 2. All catalysts exhibited C_WP_ values lower than 0.3; therefore, the results indicate the absence of mass transfer effects in ethanol decomposition reactions.

The Ni_0.7_Co_0.3_/La_0.8_Ce_0.2_ and Ni_0.4_Co_0.6_/La0_0.8_Ce_0.2_ systems exhibited a high level of conversion (≥90%) in the first hour of reaction, whereas Ni_0.1_Co_0.9_/La_0.8_Ce_0.2_ and Co/La_0.8_Ce_0.2_ showed conversions of ethanol of 74.6% and 65.4%, respectively (Table 2). These same catalysts (Ni_0.7_ and Ni_0.4_) showed higher hydrogen selectivity than Ni_0.1_ and Co catalysts. The H_2_ selectivity for Ni_0.7_Co_0.3_/La_0.8_Ce_0.2_ was 75%, whereas for Co/La_0.8_Ce_0.2_ it was only 52.6%. The fact that Ni_0.7_Co_0.3_/La_0.8_Ce_0.2_ presented higher activity and hydrogen selectivity in ethanol decomposition may be related to the dispersion, which may increase the active metal surface to participate in the transformation of ethanol.

**Figure 12:**
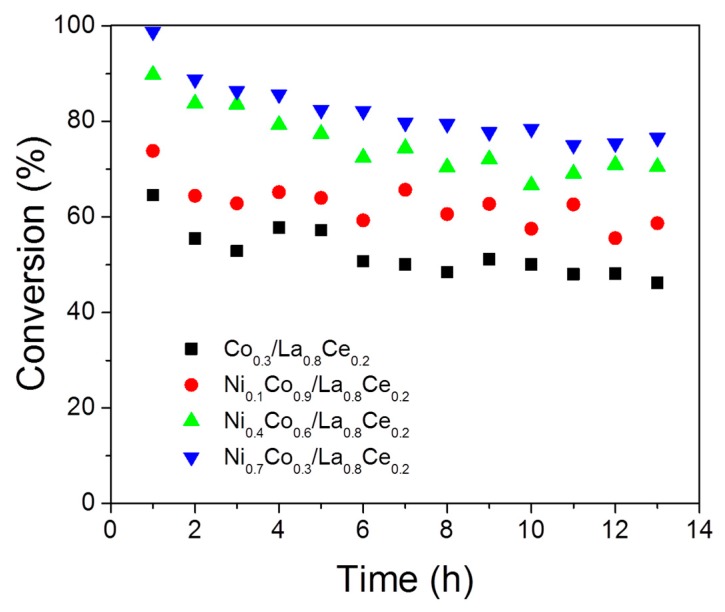
Stability tests in the reaction of ethanol decomposition over Ni_1-x_Co_x_/La_0.8_Ce_0.2_ catalysts.

Figure 12 shows the catalytic stability in the ethanol decomposition at 973 K during 13 h time on stream. The conversion level decreased slightly after 4–5 h of reaction and, afterward, the conversion of ethanol stabilized to levels of activity ≥50%. The observed decrease could be caused by the accumulation of carbon on the catalyst surface. Other products such as carbon monoxide, methane, carbon dioxide, water, and acetaldehyde were detected in the reaction of ethanol decomposition

Hydrogen, carbon monoxide, and methane were the principal products detected over the Ni_1-x_Co_x_/La_0.8_Ce_0.2_ systems. This behavior indicates that ethanol decomposition to hydrogen formation (Equation (1)) is favored at this temperature (973 K). Meanwhile, at 673 K, the systems exhibited a high production of carbon dioxide as detected by DRIFTS of ethanol decomposition. At 973 K, other compounds containing hydrogen such as methane, water, and acetaldehyde were also detected for all catalysts. The production of these compounds caused a decrease in hydrogen selectivity and their formation was related to both decomposition (Equation (1)), oxidation (Equation (4)), as well as the Boudouard (Equation (2)) and methanation (Equations (8) and (9)) reactions.

(8) $$CO+3H_{2}\;\to \;CH_{4}+H_{2}O$$

(9) $$CO_{2}+4H_{2}\;\to \;CH_{4}+2H_{2}O$$

**Figure 13:**
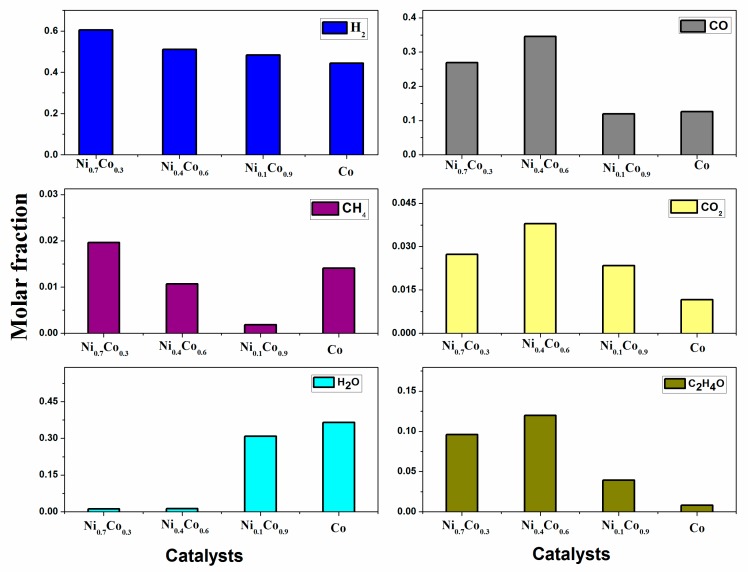
Product distribution in ethanol decomposition on Ni_x_Co_1-x_/La_0.8_Ce_0.2_ catalysts at 1 h of reaction at 973 K.

Product distribution shown in Figure 13 confirms that other reactions besides the ethanol decomposition reaction (Equation (1)) occurred on all catalysts. Ni_0.7_ and Ni_0.4_ catalysts favored the dehydrogenation route leading to acetaldehyde (Equation (4)). The highest formation of acetaldehyde on Ni_0.4_ along with the presence of acetates detected even at high temperature by DRIFTS experiments indicates that this pathway reaction can be favored on this catalyst. This fact could be responsible for the high carbon deposition observed on this catalyst (Table 3). As has been reported [22], acetaldehyde can undergo an aldol condensation reaction yielding acetone (Equation (10)), which subsequently produces diacetone alcohol. Dehydration of this species gives rise to mesityl oxide (MO) (Equation (11)), whose oligomerization can lead to the formation of coke [22]. This indicates that, on the Ni_0.4_ catalyst, the promotion of C–C bond cleavage is inefficient and the decarbonylation of acetaldehyde (Equation (6)) competes with aldol condensation and further formation of acetone. The same took place on the catalyst containing only Co, where acetate species were detected by DRIFTS until high temperatures were reached.

(10) $$2CH_{3}CHO_{\left(ads\right)}+O_{\left(s\right)}\;\to \;CH_{3}COCH_{3\left(ads\right)}+H_{2}+CO_{2}$$

(11) $$2CH_{3}COCH_{3}\;\to \;{\left(CH_{3}\right)}_{2}CO\left(OH\right)CH_{2}COCH_{3}\;\to \;{\left(CH_{3}\right)}_{2}C=CHC\left(O\right)CH_{3}+H_{2}O$$

The low amounts of CH_4_ detected in experiments carried out with the Ni_0.1_Co_0.9_ catalyst indicate that methane decomposition could be occurring, thereby promoting catalyst deactivation (Equation (3)). Furthermore, the lower amount of CO observed on Ni_0.1_Co_0.9_ and Co may be due to the participation of the Boudouard reaction (Equation (2)), which also leads to catalyst deactivation by carbon deposition. This observation agrees with obtained results by TGA and Raman experiments (see Section 3.2).

On the Ni_0.1_Co_0.9_ and Co catalysts, a high presence of H_2_O was found. H_2_O can originate from the ethanol dehydration reaction (Equation (12)), one of the most reported reactions involved in the catalyst deactivation in this type of process [2], which is favored over acid catalysts. Ethylene thus formed can polymerize to coke. According to the acidity measurements (Table 1), the catalysts with the most acidic sites were Ni_0.4_Co_0.6_/La0_0.8_Ce_0.2_ > Co/La0_0.8_Ce_0.2_ > Ni_0.1_Co_0.9_/La0_0.8_Ce_0.2_ and these were also the ones with the highest carbon content (Table 3) after 13 h of reaction in the order Ni_0.1_Co_0.9_/La0_0.8_Ce_0.2_ > Ni_0.4_Co_0.6_/La0_0.8_Ce_0.2_ > Co/La0_0.8_Ce_0.2_ Ni. Although H_2_O can also form from the methanation reaction (Equations (8) and (9)), no high CH_4_ content in evolved gases was found to justify this route.

(12) $$CH_{3}CH_{2}OH\;\to \;C_{2}H_{4}+H_{2}O$$

The Ni_0.7_Co_0.3_/La0_0.8_Ce_0.2_ catalyst displayed the higher ethanol conversion, selectivity to H_2_, and initial rate. Additionally, it was the catalyst with lower carbon deposition. These results can be related to its greater metal dispersion also associated with the highest specific surface area and much lower acidity compared to other catalysts. The amounts of metal used gave rise to an important metallic interaction, thus affecting both structural and electronic properties of the catalyst. In addition, the preparation method used led to metal nanoparticles being partially embedded in the oxide support, which displayed a lower tendency toward agglomeration and to coke deposition as mentioned in other studies [46,47,48]. Although a high carbon accumulation was observed on the catalysts, the stability only decreased slightly. This result may be due both to the formation of filamentous carbon on the catalysts, which does not always lead to catalyst deactivation [4], and to carbon gasification induced by metal–support interaction.

### 3.4. Characterization of Spent Catalysts

**Figure 14:**
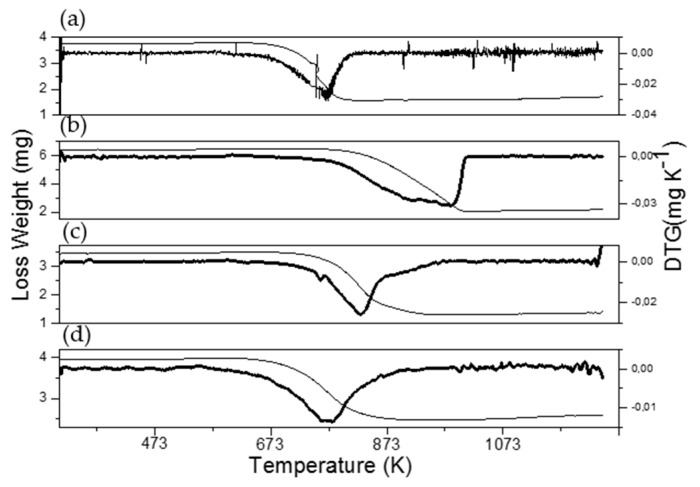
TGA-DTG of supported Ni_1-x_Co_x_/La_0.8_Ce_0.2_ catalysts after the ethanol decomposition reaction: (a) Co, (b) Ni_0.1_Co_0.9_, (c) Ni_0.4_Co_0.6_, and (d) Ni_0.7_Co_0.3_.

Thermogravimetric analyses of spent catalysts were carried out to calculate the amount of carbonaceous residues formed after ethanol decomposition by measuring the weight loss. TGA-DTG of the catalyst used containing only Co, presented in Figure 14, shows that the catalyst exhibited a peak at 770 K, corresponding to the combustion of amorphous coke [22]. Meanwhile, Ni-containing catalysts showed loss mass at higher temperatures around 800 K, which can be due to the combustion of filamentous coke with different graphitization degrees associated with nickel particles [22]. It is noteworthy that the Ni_0.1_Co_0.9_/La_0.8_Ce_0.2_ catalyst displayed a wide peak shifted at a higher temperature centered at 980 K, which can be attributed to the presence of highly ordered coke. Increasing Ni content decreased the removal temperature of carbon deposits due to its lower ordering degree obtained by particle size decreasing (Table 1). The carbon depositions for spent samples quantified by TGA are shown in Table 3. The Ni_0.7_Co_0.3_ catalyst with the smallest particle size (Table 1) was the most stable catalyst, since it showed the lowest amount of carbon deposits after 13 h of reaction, which is in agreement with previous studies [2,5,49].

**Figure 15:**
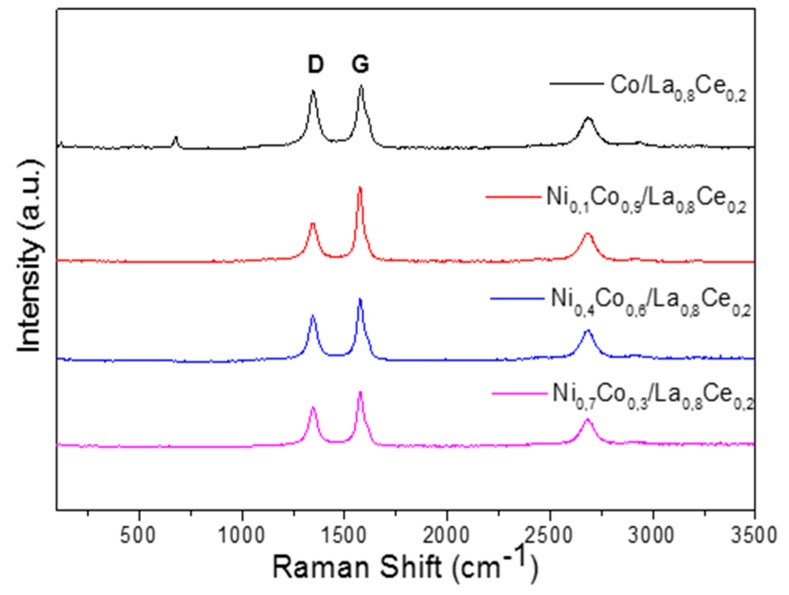
Raman spectra supported Ni_x_Co_1-x_/La_0.8_Ce_0.2_ catalysts.

On the other hand, the presence and nature of carbon deposits on the catalyst surface were evaluated by Raman spectroscopy. The results are shown in Figure 15. The bands at 1340 and 1580 cm^−1^ are labelled as D and G bands and their presence is evident in all solids. The G mode is attributed to the graphite structure, but it has also been associated in a general way with stretching vibration of C=C bond in chains or in aromatic rings, whereas the D mode is the breathing mode of C=C bonds only in rings [50,51]. This band is associated with the Raman mode of disordered defective carbon structures. Therefore, the heterogeneity of the carbon species can be related to the presence of both signals, G and D. The intensity ratio of the D and G Raman bands (ID/IG) gives information on the type of coke [50] prevailing during reaction. For Ni_0,1_Co_0,9_/La_0,8_Ce_0,2_, a lower value was obtained, which agrees with the TGA results where a higher temperature was necessary to remove carbon deposits, as is shown in Figure 12 and Table 3.

## 4. Conclusions

Co-Ni bimetallic catalysts supported on La-Ce oxides were prepared by reducing La_0.8_Ce_0.2_Ni_1-x_Co_x_O_3_ perovskite precursors. The preparation method resulted in very small particle sizes that diminished with nickel content increase in the order Ni_0.7_Co_0.3_/La0_0.8_Ce_0.2_ ≤ Ni_0.4_Co_0.6_/La0_0.8_Ce_0.2_ < Ni_0.1_Co_0.9_/La0_0.8_Ce_0.2_ < Co/La0_0.8_Ce_0.2_ according to H_2_ chemisorption and TEM measurements. The Ni_0.7_Co_0.3_/La0_0.8_Ce_0.2_ catalyst exhibited the higher ethanol conversion (98.4%) and selectivity to H_2_ (75%) as well as the lower carbon deposit formation during the ethanol decomposition reaction as determined by TGA measurements. These observations were attributed to its smaller size particle, higher specific surface area, and much lower acidity compared to other catalysts. Despite the presence of coke, all catalysts were stable for 13 h of time on stream, which can be related to the strong metal–support interaction obtained due to the use of perovskites as precursors for the catalysts. This interaction can increase carbon gasification and decrease metal sintering as reported previously. Several side reactions are involved in H_2_ production by ethanol decomposition that can favor catalyst deactivation. According to product distribution, the side reactions responsible for the formation of carbon deposits were mainly methane decomposition, Boudouard reaction, and ethanol dehydration. In addition, acetate species could have blocked the active sites as observed in DRIFTS experiments, contributing also to the deactivation of the catalysts. Finally, the preparation method used in this study led to bimetallic Ni-Co catalysts supported on La_2_O_3_-CeO_2_ with small particle sizes, as well as being active and stable for ethanol decomposition for H_2_ production.

## Figures and Tables

**Table 1:** Structural parameters of supports and Ni_1-x_Co_x_/La_0.8_Ce_0.2_ catalysts.

Catalysts	Surface Area BET (m^2^ g^−1^)	Pore Volume (cm^3^ g^−1^)	Pore Size (nm)	Dispersion (%)	Particle Size ^a^ (nm)	Particle Size ^b^ (nm)	Acidity (mol NH_3_ g_cat_^−1^)
Ni_0.7_Co_0.3_/La_0.8_Ce_0.2_	33	0.13	25	21	4.8	9.1	0.04
Ni_0.4_Co_0.6_/La0_0.8_Ce_0.2_	16	0.05	28	10	10	5.9	0.47
Ni_0.1_Co_0.9_/La_0.8_Ce_0.2_	14	0.04	31	6	17	12.4	0.23
Co/La_0.8_Ce_0.2_	23	0.06	45	3	33	16.8	0.35

^a^ Determined by H_2_ chemisorption; ^b^ Determined by TEM.

**Table 2:** Initial rate of reaction, turnover frequency (TOF), Weisz–Prater criterion, conversion, and hydrogen selectivity for ethanol decomposition. Reaction conditions: total flow, 100 mL min^−1^ (5% ethanol; 95% He); temperature of reaction, 973 K; catalyst weight, 100 mg at 1 h of reaction.

Catalysts	(−*r_a_*)_0_ (mol g^−1^ s^−1^)	TOF (s^−1^)	C_WP_	Ethanol Conversion (%)	Hydrogen Selectivity (%)
Ni_0.7_Co_0.3_/La_0.8_Ce_0.2_	6.2 × 10^−4^	0.06	6.3 × 10^−3^	98.4	75.0
Ni_0.4_Co_0.6_/La0_0.8_Ce_0.2_	5.6 × 10^−4^	0.11	2.8 × 10^−3^	90.0	64.5
Ni_0.1_Co_0.9_/La_0.8_Ce_0.2_	4.7 × 10^−4^	0.15	2.8 × 10^−3^	74.6	56.3
Co/La_0.8_Ce_0.2_	4.1 × 10^−4^	0.27	1.5 × 10^−3^	65.4	52.6

**Table 3:** Carbon deposition on Ni_x_Co_1-x_/La_0.8_Ce_0.2_ catalysts evaluated by TGA.

Catalyst	mg_coke_/g_cat_	T_peak_ DTG	I_D_/I_G_
Co/La_0.8_Ce_0.2_	530.5	773	1.00
Ni_0.1_Co_0.9_/La_0.8_Ce_0.2_	659.5	980	0.98
Ni_0.4_Co_0.6_/La_0.8_Ce_0.2_	592.1	830	0.99
Ni_0.7_Co_0.3_/La_0.8_Ce_0.2_	342.6	780	0.99
